# The association between rheumatoid arthritis and periodontal disease in a population-based cross-sectional case-control study

**DOI:** 10.1186/s41927-020-00129-4

**Published:** 2020-07-20

**Authors:** Stefan Renvert, Johan Sanmartin Berglund, G. Rutger Persson, Maria K. Söderlin

**Affiliations:** 1 Faculty of Health Sciences, Kristianstad University, SE-291 88 Kristianstad, Sweden; 2 Department of Health, Blekinge Institute of Technology, SE-371 79 Karlskrona, Sweden; 3 School of Dental Science, Trinity College, Dublin, Ireland; 4 Faculty of Dentistry, The University of Hong Kong, Hong Kong, SAR China; 5 Departments of Periodontics, and the Department of Oral Medicine, University of Washington, Seattle, WA USA; 6 Department of Clinical Sciences, Section of Rheumatology, Lund University, Lund, Sweden

**Keywords:** Rheumatoid arthritis, Periodontitis, Epidemiology, Smoking

## Abstract

**Background:**

The association between rheumatoid arthritis (RA) and periodontitis remains unclear.

**Methods:**

We studied oral health and periodontitis in a population-based case-control study of individuals with ≥10 remaining teeth ≥61 years of age and either with, or without a diagnosis of RA. 126 dentate individuals with RA were recruited together with age-matched control individuals without RA. The control individuals were recruited from the general population from the same city (*n* = 249). A dental examination including a panoramic radiograph was performed on all participants. All individuals with RA were examined and medical records were reviewed by a rheumatologist. In the control group, none of the participants presented with symptoms of RA and their medical records were also negative.

**Results:**

The RA group included more women (66.7% vs. 55.8%) (*p* < 0.01). Individuals in the RA group had a higher body mass index (BMI) (*p* < 0.001). A diagnosis of periodontitis was more common in the RA group (61.1%) than in the control group (33.7%) (*p* = 0.001). Binary logistic regression analysis identified that a BMI > 25 (OR 6.2, 95% CI 3.6, 10.5, *p* = 0.000), periodontitis (OR 2.5 95% CI 1.5, 4.2 *p* = 0.000), and female gender (OR 2.3, 95% CI 1.3–4.0, *p* = 0.003) were associated with RA.

**Conclusion:**

RA was associated a diagnosis of periodontitis.

## Background

Rheumatoid arthritis (RA) is a chronic inflammatory disease with multifactorial etiologies [1]. RA is a complex disease involving genetic, autoimmunity, infection, gender, smoking, and environmental factors [2–5]. Several other conditions have been identified as comorbidities to RA including cardiovascular disease, malignancies and osteoporosis [6].

The infectious etiology of periodontitis is well established [7, 8]. Genetic predisposition has also been associated with infectious susceptibility in subjects with periodontitis [9]. Although RA is a disease of the joints, and periodontitis a disease of the oral soft tissues and alveolar bone, both disease entities include chronic inflammation resulting in connective tissue breakdown and bone erosion.

Recent data suggest that the HLA-DRB1 genetic locus is strongly associated with susceptibility to RA through citrullinated self-peptides binding to HLA-DR molecules [10]. *Porphyromonas gingivalis* is considered as a critical pathogen in periodontitis, and the only currently known human pathogen expressing peptidyl arginine deiminase [11–13]. This enzyme generates citrullinated epitopes recognized by anti-citrullinated protein antibodies which has been linked to RA [14]. In one prospective study, periodontitis was more common among individuals with RA who also had higher levels of *Porphyromonas gingivalis* in deeper periodontal pockets than found in control individuals [15]. At the follow-up examinations, individuals with RA receiving treatment of the disease with a disease-modifying antirheumatic drug (DMARD) still showed poor periodontal conditions [15].

The association between periodontitis and RA has been studied in several studies [15–24]. In some studies, a high prevalence of periodontitis, and tooth loss in individuals with RA has been identified [16, 21, 22]. In contrast, data from the National Health and Nutrition Survey (NHANES I) suggest that although individuals with periodontitis, or ≥ 5 missing teeth experienced higher odds of prevalence/incidence of RA, most odds ratios published were non-statistically significant [25].

The objective of the present population-based cross-sectional study was to assess if a diagnosis of periodontitis is more common in individuals (≥ 61 years) with RA than among age-stratified individuals from the normal population without a diagnosis of RA.

## Methods

The study complies with the Declaration of Helsinki. The Regional Ethical Review Board at Lund, Sweden, approved the study (LU 2013/323). The study individuals gave their informed consent to participate in the study. All study individuals received their dental and medical assessments between 2013 and 2015.

### Selection of study individuals

**Fig. 1:**
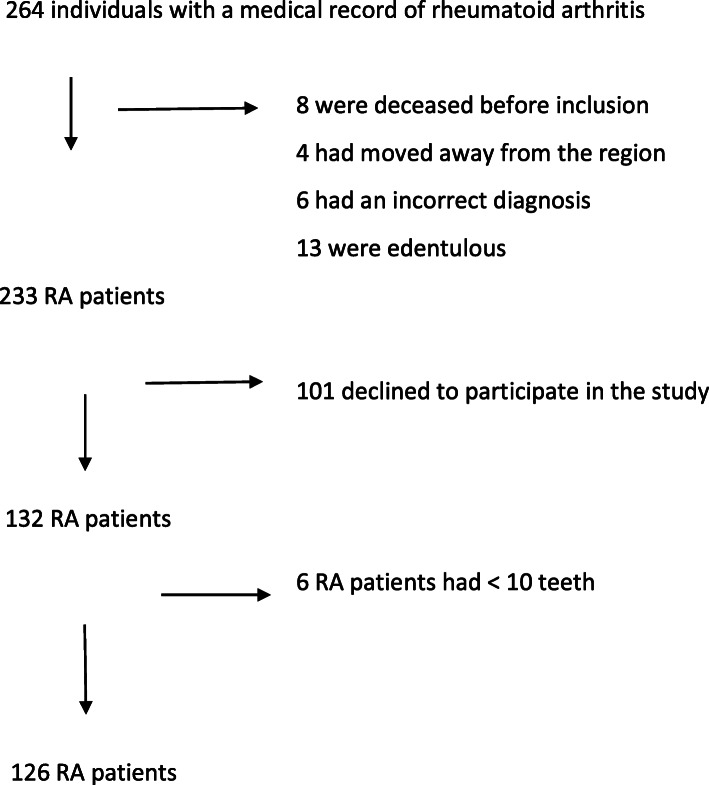
264 individuals with a medical record of rheumatoid arthritis

Individuals in the RA group were identified from medical electronical records at the regional hospitals of Region Blekinge (population 153,000 in 2013). To be included in the study, RA patients (M05 and M06, International Classification of Diseases ICD-10) had to be > 61 years of age and living in Karlskrona city (population 64,000). A total of 233 individuals age ≥ 61 diagnosed with RA were identified and invited to participate in the study. Classification of the RA patients was performed according to the 1987 ACR RA criteria [26] and the 2010 ACR/EULAR classification criteria [27]. Consent to participate was given by 132 individuals with RA. Following the dental examinations only those individuals with ≥10 remaining teeth were included which resulted in 126 study participants with a diagnosis of rheumatoid arthritis. The study enrollment flow chart is presented in Fig. 1. The age of > 61 years in the RA patients was chosen to be able to match the RA patients with controls (see below).

All individuals with RA were examined at the outpatient rheumatology clinic by rheumatologists. Medical records of the RA patients and controls were also reviewed by a rheumatologist (author MS). Data on RA disease activity, and current antirheumatic medications at inclusion were identified at the rheumatologists’ visit and confirmed from the Swedish Rheumatology Quality Register online (www.srq.se). Data on disease duration, previous anti-rheumatic medications, comorbidities, osteoporosis, smoking habits, occupation, body mass index (BMI), the total number of drugs and blood analysis including cholesterol levels, rheumatoid factor were recorded.

The individuals in the control group were chosen among 1101 dentate individuals participating in the ongoing Swedish Study on Aging and Care study in Blekinge (SNAC - Blekinge). The SNAC study is a longitudinal cohort study including individuals from the normal population who at the time of enrolment were 60 years of age or older (www.snac.org). Based on age characteristics two age-matched individuals (control group) were identified from the SNAC study to each individual with RA. The age matching was performed such that one control individual with a birth date within a few months before the RA individual, and one control individual with a birth date within a few months after the RA individual were selected. The range of age of the controls was 60–89. In the control group, none of the participants presented with symptoms of RA and their medical records were also negative. Thus, these control individuals were defined as not having a diagnosis of RA. All control individuals must also have ≥10 remaining teeth. A total of 249 control individuals were included in the study.

### Demographics and health characteristics

Information on socio-economic status (white/blue collar workers), smoking history, and diabetes was obtained. Smoking status defined as current/past smoker or never smoker. Overweight was defined as BMI > 25. Data from medical records were assessed to identify whether a diagnosis of cardiovascular diseases, stroke, interstitial lung disease, osteoporosis, or other diseases were associated with RA or periodontitis. In the statistical analyses of these factors dichotomized data were used.

### Dental examinations

A dental hygienist performed the clinical dental examinations of all study individuals. The examination also included a panoramic radiograph. Radiographs were assessed for the number of remaining teeth, measurements of alveolar bone levels, and identification of other dental conditions. The radiographic assessments were performed by a periodontist (author GRP) who was unaware of the participants´ medical conditions.

The following dental examinations were performed:

1. Measurements of probing pocket depths at four surfaces at all teeth and dental implants (mesial, buccal, distal, lingual/palatal).
2. The extent of bleeding on probing (BOP) was recorded within 30 s after probing of probing pocket depth measurements and assessed at the same four surfaces as above.
3. Dental plaque scores were assessed at the same four surfaces (mesial, buccal, distal, lingual/palatal) at all teeth and dental implants.
4. Tooth decay was recorded as open decay.
5. The presence/absence of abnormal mucosal conditions was recorded.
6. The number of remaining teeth was registered and confirmed from the radiographs.
7. The extent of alveolar bone loss (mm distance between the cement-enamel junction (CEJ) and bone level at interproximal sites) was assessed from panoramic radiographs. The proportions of sites with a depth ≥ 4 mm and ≥ 5 mm in relation to the number of assessed interproximal sites were calculated to arrive at a subject based number defining bone loss. The distances were assessed using digital images and the Osirix software version 9.0 (Pixmeo, SARL, Bernex, Switzerland).

### Definition of gingivitis and periodontitis

Gingivitis was defined as having ≥20% of measured sites with evidence of bleeding on probing. Periodontitis was defined as the clinical presence of bleeding on probing at > 20% of recorded tooth surfaces, presence of > 2 non-adjacent sites with a probing pocket depth (PPD) ≥ 5 mm, presence of bone loss at ≥2 sites with a distance between cement enamel junction-to bone level of ≥5 mm, or if evidence of a furcation invasion at molar teeth was found either clinically (grade II), or clearly visible on panoramic radiographs. The parameters for gingivitis and periodontitis were used in a dichotomous composite periodontal index. All study individuals must have at least 10 remaining teeth.

### Statistics

Independent T-tests for continuous variables (equal variance not assumed) were performed to compare data between individuals in the RA and control groups. The Mann-Whitney test was used for non-parametric data. Chi-square analyses were used for dichotomous variables. The radiographs were read twice. The results from intraclass correlation were analysed to define the precision of the radiographic readings. The data were also analyzed using backward logistic binary regression analysis including variables known to be linked to RA and periodontitis. Statistical analyses were performed using IBM SPSS Statistics version 25 (Armonk NY, USA).

## Results

Data from 126 individuals with RA were analyzed giving a catchment of 54%. The 110 RA patients who declined to participate in the study were older (74 vs. 70, *p* = 0.0001), had a higher mean erythrocyte sedimentation rate (ESR) (27 vs. 19, *p* = 0.0001), had a higher mean DAS28ESR (Disease Activity Score using ESR) (3.4 vs. 3.0, *p* = 0.005) and were less often on biologics (7% vs. 22%, *p* = 0.003), but did not otherwise differ from the RA patients included in the study. A total of 83% of the RA patients fulfilled the 1987 ACR classification criteria and 71% the 2010 ACR/EULAR classification criteria for RA. The period prevalence of RA was 1.5%.

### Demographics and health characteristics of the study population

Demographics and health characteristics of the study population are summarized in Table 1.

**Table 1:** Demographics and health characteristics of the study population. * data missing in some cases

Variable	RA Group *N* = 126	Control group *N* = 249	*P* value
Female %	70%	56%	0.009
Age, mean (SD)	70 (6.6)	70 (7.1)	0.39
Smoking habit %		*N* = 234*	0.58
Current/past smokers N (%)	78 (61.9%)	140 (59.8%)	
Socio-economic status (SES)			
White collar	47.6%	54.2%	0.75
Blue collar	52.4%	45.8%	
BMI, mean (SD)	27 (5.1)	23 (3.7)	0.000
Diabetes (including both type I and II) N (%)	17 (13.5%)	20 (8.1%)	0.10
Interstitial lung disease N(%)	5 (4.0%)	0 (*N* = 240*)	0.002
Angina pectoris N (%)	15 (11.9%)	26 (10.5%) *N* = 247*	0.69
Myocardial infarct N (%)	10 (7.9%)	15 (6.0%) *N* = 248*	0.49
Stroke N (%)	11 (8.7) %	10 (4.0%) *N* = 248*	0.06

*BMI* body mass index

All study individuals in the RA group were born in Sweden. The study group with a diagnosis of RA included more women than in the control group (*p* < 0.01). The mean BMI was higher in the RA group (*p* < 0.001). Interstitial lung disease was more common in individuals with RA (*p* = 0.002). Analyses failed to demonstrate study group difference by socio-economic status (SES) (*p* = 0.72), or for a history of smoking (*p* = 0.58). The age matching criteria were successful in recruiting individuals of similar ages (both with a mean value of 70 years) for the two study groups (*p* = 0.39).

### Rheumatoid arthritis

Table 2 describes the disease activity assessments, and antirheumatic medications for the study individuals with RA. Among the individuals with RA, 66% were on DMARDs (disease-modifying antirheumatic drugs), 57% were using methotrexate, 22% were using biologics and 45% were medicated with glucocorticoids. A majority of the individuals in the RA group (63%) were in remission or diagnosed as having low disease activity. Using ≥25 U/mL anti- CCP-2 (antibodies against citrullinated proteins) analysis in serum, 66% of the study individuals were positive.

**Table 2:** Disease activity variables for individuals with RA

Variable	N	
Disease duration from symptom start, years, mean (SD)	121	14 (13)
VAS pain, mm, mean (SD)	123	35 (28)
ESR, mm, mean (SD)	123	19 (15)
CRP, mg/ml, mean (SD)	126	9 (8)
DAS28ESR classes, %	125	
DAS28 < 2.6 remission		40.5%
2.61–3.2 low disease activity		22.2%
3.21–5.1 moderate disease activity		31%
> 5.1 high disease activity		5.6.%
Anti - CCP positive %	124	66%
RF positive %	123	56%
Number of current DMARDs, mean (SD)	126	0.8 (0.6)
Total number of all medications at inclusion, mean (SD)	126	9.9 (4.3)

*NA* not available, *VAS* visual analogue scale, *ESR* Erythrocyte sedimentation rate, *CRP* C- reactive protein, *DAS28ESR* Disease Activity Score (28 joints) calculated with ESR, *Anti-CCP* Anti-cyclic citrullinated protein, *RF* Rheumatoid factor, *DMARD* Disease-modifying anti-rheumatic drug

### Findings from the dental examination

Data from the dental examination are presented in Table 3. Radiographic evidence of bone loss (distance between CEJ to bone ≥5 mm) was more common in the RA-group (*p* = 0.03). The number of remaining teeth was higher in the control group (*p* = 0.001). No significant group differences were, however, found for the number of sites with a PPD value ≥5 mm, BOP, or plaque scores, the number of root remnants, or peri-apical lesions. Data analyses were also unable to show group differences for the number of teeth with open carious lesions (*p* = 0.30), or for the number of dental implants present. In addition, no group differences were found regarding the number of dental visits per year (94% vs 90%, *P* = 0.30). A dental diagnosis of periodontitis based on the composite periodontal index was higher in the RA group (*p* < 0.001).

**Table 3:** Dental characteristics of study individuals in the RA and control groups

**Definition**	**RA group**		**Control group**		***P*value**
Periodontitis yes/no (% yes)	***N*** **= 126**		***N*** **= 249**		0.001
	61/65 (48%)		96/153 (38.6%)		
	**Mean**	**SD**	**Mean**	**SD**	***P*value**
Number of remaining teeth	22.	4.6	24.5	4.3	0.001
Number of teeth with endodontic treatment	3.2	3.2	3.1	2.5	0.72
Number of peri-apical lesions	0.3	0.7	0.3	0.6	0.97
Root remnants	0.2	1.0	0.5	6.2	0.59
BOP % (present at ≥20% of sites assessed)	24.5	22.6	21.8	16.8	0.24
Plaque score % (present at ≥20% of sites assessed)	31.5	23.9	32.4	21.9	0.54
Number of sites with a PPD ≥ 5 mm	3.3	6.2	2.7	5.4	0.36
Bone level ≥ 5 mm at ≥30% sites examined radiographically (% sites)	18.7	23.5	13.5	7.6	0.03

*PPD* probing pocket depth, *BOP* bleeding on probing

### Subset analysis of periodontal conditions by gender among individuals in the RA group

There were no gender differences regarding the number of remaining teeth (*p* = 0.81), the extent of bleeding on probing (*p* = 0.65), the number of tooth sites with a probing depth ≥ 5 mm (*p* = 0.98), or the bone level distance ≥5 mm (*p* = 0.75). Also, no gender difference in the presence or absence of periodontitis was found (*p* = 0.92).

Treatment with methotrexate, DMARDs, biologics, glucocorticoids and non-steroidal anti-inflammatory drugs (NSAIDs) had no impact on periodontal conditions (*p* values varied between 0.70 and 0.95).). A sub analysis on RA patients with a positive or negative anti-CCP titer was also performed. In this sub analysis no association between anti-CCP positivity and periodontitis was found (*p* = 0.92).

### Subset analysis of periodontal conditions by gender among individuals in the control group

There were no gender differences regarding the number of remaining teeth (*p* = 0.49), the extent of bleeding on probing (*p* = 0.65), the number of tooth sites with a probing depth ≥ 5 mm (*p* = 0.12), or the bone level distance ≥5 mm (*p* = 0.18). Also, no gender difference in the presence or absence of periodontitis was found (*p* = 0.68).

### Analyses by binary backward conditional logistic regression

Based on information from the literature and from the results presented above the following categorical variables were included: gender, acute myocardial infarct, stroke, diabetes (I and II merged), BMI score > 25, white collar or blue collar worker (SES), smoking history (never or anytime), and age. In addition, periodontitis as defined above by the composite index. In the analysis, bleeding on probing was defined as the expression of gingivitis ≥20% of sites, probing pocket depth ≥ was defined at 3 sites or more with a PPD score ≥ 5 mm and alveolar bone loss was defined as a distance between CEJ to bone ≥5 mm at ≥30% of sites.

The following covariates remained in the final analysis; BMI > 25, gender, composite periodontal score (Table 4). Other covariates including age (*p* = 0.80), acute myocardial infarction (*p* = 0.81), stroke (*p* = 0.23), socio-economic status (*p* = 0.77) and smoking status (*p* = 0.73) failed to reach significance.

**Table 4:** Results (final step) from the binary backward conditional logistic regression analysis

Variables	Binary logistic regression		
	Odds ratio	95% CI:	Sign.
BMI > 25	6.2	3.6–10.5	*P* = 0.000
Periodontitis (composite index)	2.5	1.5–4.2	*P* = 0.000
Gender (female)	2.3	1.3–4.0	*P* = 0.003

*BMI* body mass index

## Discussion

In this population-based cohort on elderly RA patients we found that RA was associated with periodontitis with an OR of 2.5. This finding is in contrast to the data reported in the study from the Swedish Quality Registry for Caries and Periodontal Diseases [28], which is based on clinical patient records collected from a large number of public and private dental offices, and from the Swedish Epidemiological Investigation of RA [18, 19]. No evidence of an increased prevalence of periodontitis in patients with established RA compared to healthy controls could be found in this Epidemiological Investigation of Reumatoid Arthritis (EIRA) study [18, 19]. Data analyses from this EIRA registry study, however, suggests that smoking and aging are risk factors for periodontitis, both in RA and controls.

Other recent studies have also identified that periodontitis is associated with RA [22, 29]. It should be recognized that such an association does not suggest a direct causal relationship.

Over time, several different criteria and classification methods of periodontitis have been published [30–35]. The present study was designed before the latest revision of the principles, and criteria for periodontal diseases were published [35, 36]. In the present study, we have focused on evidence of gingival inflammation, evidence of probing pocket depth, clinical evidence of furcation invasion at molars, and radiographic evidence of alveolar bone levels exceeding a defined threshold value to assess the association to RA. Our composite definition of periodontitis is, in principle, consistent with the current staging definition system (stage I to IV of periodontitis).

In the present study, the presence of elevated levels of bleeding on probing, or the presence of pathological pockets as identified, could not independently identify differences between individuals with or without RA. In contrast, the composite periodontal index used in the present study, identified that periodontitis as defined was associated with RA. The variables included in the composite periodontal index that we used reflect both present and past signs and effects of inflammation reflecting also the accumulated effects of inflammatory host responses resulting in periodontitis. It may seem contradictory when current signs i.e. bleeding on probing, or probing pocket depths were excluded by the statistical model.

The strength of the present population-based study was the systematically sampled population-based elderly RA cohort and a well-balanced systematically recruited group of control individuals from the normal population. In most other clinical studies on RA and periodontitis, the RA population has been recruited from the rheumatology departments, but not in a population-based way, and the controls have been chosen from either other patients or health care workers, and not representative of a normal population. The RA diagnosis in the present study was clinical, while some other studies have used RA patients fulfilling the 1987 ACR and /or 2010 ACR/EULAR criteria. The majority of the RA patients in the present study were stable and treated according to standard of care for RA. The prevalence of periodontitis based on the diagnostic criteria used for periodontitis in the present study suggest a rather low prevalence in comparison to other studies. It should, however, be highlighted that the definition used for periodontitis may have selected individuals with rather advanced disease. It was not possible to identify the cause of tooth loss. Approximately 90% of the study individuals from both study groups received regular dental care, which could be the reason for the relatively low frequency of periodontitis reported. Access to low cost high quality of dental care has been available in the geographical area from which the study participants were recruited.

One limitation of the present study was that approximately 50% of the identified group of individuals with RA either declined participation or they did not meet the dental inclusion criteria. One of the explanations why various studies have either identified or failed to identify associations between RA and periodontal conditions is most likely problems associated with how to define RA and periodontitis and the recruitment of RA patients and choice of controls. Another study limitation was that information on periodontal treatments and causes for tooth extractions was not available. Furthermore, we did not have information on whether the study participants had previously been treated for periodontitis. Since the individuals were not matched according to BMI it is possible that the difference in BMI between groups may have influenced the association between RA and periodontitis.

## Conclusion

The present study identified that rheumatoid arthritis was associated with a diagnosis of periodontitis.

## Data Availability

Please contact author for data requests.

## Abbreviations

ACR: American College of Rheumatology
BMI: Body mass index
BOP: Bleeding on probing
CCP: Cyclic citrullinated peptide
CEJ: Cement enamel junction
DAS28ESR: Disease Activity Score using erythrocyte sedimentation rate
DMARD: Disease-modifying antirheumatic drug
EIRA: Epidemiological Investigation of Reumatoid Arthritis
ESR: Erythrocyte sedimentation rate
EULAR: European League Against Rheumatism
ICD: International Classification of Diseases
LU: Lund University
PPD: Probing pocket depth
RA: Rheumatoid arthritis
SES: Socio-economic status
SNAC: Swedish Study on Aging and Care
